# Gut Transcription in *Helicoverpa zea* is Dynamically Altered in Response to Baculovirus Infection

**DOI:** 10.3390/insects4030506

**Published:** 2013-09-23

**Authors:** Jeffrey E. Noland, Jonathan E. Breitenbach, Holly J. R. Popham, Sue M. Hum-Musser, Heiko Vogel, Richard O. Musser

**Affiliations:** 1 Department of Biological Sciences, Western Illinois University, Waggoner Hall 358, Macomb, IL 61455, USA; E-Mails: jeffrey.noland@uky.edu (J.E.N.); SM-Hum-Musser@wiu.edu (S.M.H.-M.); 2 USDA Agricultural Research Service, Arthropod-Borne Animal Diseases Research Unit, 1515 College Avenue, Manhattan, KS 66502, USA; E-Mail: Jonathan.Breitenbach@ars.usda.gov; 3 USDA Agricultural Research Service, Biological Control of Insects Research Laboratory, 1503 S. Providence, Columbia, MO 65203, USA; E-Mail: Holly.Popham@ars.usda.gov; 4 Department of Entomology, Max Planck Institute for Chemical Ecology, Beutenberg Campus, Jena 07745, Germany; E-Mail: hvogel@ice.mpg.de

**Keywords:** *Helicoverpa zea*, HzSNPV, gut epithelium, microarray

## Abstract

The *Helicoverpa zea* transcriptome was analyzed 24 h after *H. zea* larvae fed on artificial diet laced with *Helicoverpa zea* single nucleopolyhedrovirus (HzSNPV). Significant differential regulation of 1,139 putative genes (*p* < 0.05 T-test with Benjamini and Hochberg False Discovery Rate) was detected in the gut epithelial tissue; where 63% of these genes were down-regulated and 37% of genes were up-regulated compared to the mock-infected control. Genes that play important roles in digestive physiology were noted as being generally down-regulated. Among these were aminopeptidases, trypsin-like serine proteases, lipases, esterases and serine proteases. Genes related to the immune response reacted in a complex nature having peptidoglycan binding and viral antigen recognition proteins and antiviral pathway systems down-regulated, whereas antimicrobial peptides and prophenoloxidase were up-regulated. In general, detoxification genes, specifically cytochrome P450 and glutathione S-transferase were down-regulated as a result of infection. This report offers the first comparative transcriptomic study of *H. zea* compared to HzSNPV infected *H. zea* and provides further groundwork that will lead to a larger understanding of transcriptional perturbations associated with viral infection and the host response to the viral insult in what is likely the most heavily infected tissue in the insect.

## 1. Introduction

Interactions between insects and the viruses that exploit them as part of their replicative cycle offer us among the oldest examples of infectious outcomes in the animal kingdom. Baculoviruses are thought to have diverged from nudiviruses approximately 350 MYA, and baculovirus-infected insects can be found all over the globe [1,2,3]. The study of baculoviruses has both offered us information regarding the insect response to infection, and an exploitable means of controlling populations of susceptible pest insects [4,5,6,7,8]. Indeed, commercialized baculovirus compositions have been labeled for use as bio-insecticides since the late 1970s, and are seeing resurgence in many parts of the world [9,10].

Baculoviridae are divided into alphabaculoviruses, betabaculoviruses, gammabaculoviruses, and deltabaculoviruses [11]. Alphabaculoviruses, the topic of this study, either incorporate one (single nucleopolyhedrovirus) or more (multiple nucleopolyhedrovirus) nucleocapsids within a viral envelope, embedded within electron-dense crystal structures termed polyhedra [12,13]. Following ingestion, polyhedra are exposed to the alkaline pH of the larval gut resulting in its dissolution and release of baculovirus virions [14]. Contact between the viral envelope glycoprotein and the apical surface of gut cells is thought to promote viral entry and establishment of primary infection. The baculovirus replication cycle proceeds in a coordinated and temporal fashion, with transcription of immediate early genes, followed by early genes that promote and facilitate DNA replication, followed by late genes that give rise to proteins responsible for packaging daughter genomes in capsids, and those responsible for complete assembly, maturation and egress of the virus [14,15,16]. Dissemination of the virus results in secondary and subsequent rounds of infection, leading to polyhedra formation, liquefaction of host tissues, enzymatic digestion of the exoskeleton chitin, and release of the viral polyhedra into the surrounding environment, thus driving spread of the virus to other host insects [14,15,16]. 

Much effort by numerous groups has gone into identifying, defining, and extending our knowledge regarding the function of individual baculovirus genes and groups of host genes that are specifically responsive to baculovirus infection [17,18,19,20]. However, microarray analysis, and more recently high-throughput RNA-seq approaches, offer us new means to examine the transcriptional responses of both the virus and host in non-model species, on a far more broad basis [20,21,22,23,24,25,26,27]. In this report, we examine by microarray technology the transcriptional profile of the gut of *Helicoverpa zea* larvae (Lepidoptera: Noctuidae) either mock-infected or experimentally challenged with a lethal dose of the alphabaculovirus *Helicoverpa zea* single nucleopolyhedrovirus (HzSNPV). We validate the DNA microarray results with quantitative real-time PCR (qRT-PCR) to confirm the observed up-regulation/down-regulation of specific genes, and finally, we offer ontological analysis of groups of differentially regulated genes.

## 2. Experimental Section

### 2.1. Insect Rearing, Infection, and Gut Isolation

*H. zea* larvae were grown from neonates on a corn based artificial diet (BioServ, Frenchtown, NJ, USA) in separate medicine cups (Solo Cup Company, Lake Forest, IL, USA) at 33 °C until the first day of the 6th instar. The larvae were starved for two hours and then fed a 2.0 cm^3^ cube of artificial diet coated with either 7.5 µL of double-distilled water (mock) or an HzSNPV Elcar inoculum (Thermo Trilogy Corp, Columbia, MD, USA) containing ~1,500 occlusion bodies (OBs) in 7.5 µL of double-distilled water. The larvae were allowed to feed for 24 h after which the gut from each larva was excised and the peritrophic matrix was removed, thus isolating the gut epithelium. Four biological replicates were collected each consisting of eight larval gut epithelial tissues. 

### 2.2. Total RNA Isolation

Gut epithelial tissues were immediately placed in 1.0 mL of TRIzol (Life Technologies, Carlsbad, CA, USA), ground until the tissue was homogenized, and total RNA was purified and recovered in 100 µL of nuclease free water according to the manufacturer’s protocol. The purity and concentration of the RNA was measured with a Nanodrop 2000 UV-VIS Spectrophotometer (Agilent Technologies, Santa Clara, CA, USA) using samples with a A260/280 purity ratio of 2.0 ± 0.1. RNA was stored at −80 °C pending further processing.

### 2.3. Amplification and Labeling of mRNA

Amplification and labeling of the mRNA was completed using the Agilent two-color Low Input Quick Amp Labeling Kit (Agilent Technologies, Inc., Santa Clara, CA, USA). To summarize, 200–400 ng of total RNA was added with a T7 Promoter Primer for cDNA synthesis, then the labeled cRNA was synthesized and purified. cRNA and dye concentrations were determined by spectrophotometry. Specific activity of labeled cRNA was determined according to the manufacturer’s protocol and a specific activity of at least 8 OD was used for microarray analysis.

### 2.4. Hybridization and Scanning of the Microarray Chip

*Helicoverpa armigera* microarray slides (Agilent Technologies) used for transcriptome profiling were developed by Dr. Heiko Vogel (Max Plank Institute for Chemical Ecology, Jena, Germany) [28]. Each microarray had four arrays per chip, each containing 44,000 oligonucleotides with approximately 27,000 putative gene targets. Following Agilent protocols, samples were added with fragmentation and hybridization buffers and pipetted onto a gasket slide covering each Agilent array. The chip and cRNA samples were incubated together at 65 °C for 17 h. Following hybridization, the microarray chips were treated twice with Gene Expression (GE) wash buffer and once with GE Wash Buffer 2 containing 0.01% wash buffer additive according to the manufacturer’s protocol. The slides were then dipped in an acetonitrile dip and Agilent Drying Solution to prevent oxidation by ozone.

The arrays were then immediately scanned with a GenePix Personal 4100 Microarray Scanner (Axon Instruments Inc., Forster City, CA, USA) following the protocol described by Musser *et al*. (2012) [29]. The arrays were scanned at 5.0 µm to elucidate the best image with photomultiplier tube (PMT) gain adjusted to maximize the total number of features to measure and was normalized on a genomic scale for the dyes to have a 1:1 ratio and further normalized using GeneSifter software (Geospiza, PerkinElmer, Inc.) [30].

### 2.5. Microarray Experimental Design and Analysis

To detect and quantify gene expression, samples were arranged as four biological replicates on microarrays and dyes were reversed using a replicated loop design (6) as follows: array one—Virus 4 (Cy-5) *vs*. Control 2 (Cy-3), array two—Virus 3 (Cy-5) *vs*. Control 1 (Cy-3), array three—Virus 2 (Cy-3) *vs*. Control 4 (Cy-5), and array four—Virus 1 (Cy-3) *vs*. Control 3 (Cy-5). Data collected from each experiment was uploaded to the GeneSifterTM software. The data was log-transformed and scaled across all microarrays. A Benjamini-Hochberg corrected two-sample *t*-test (*p* < 0.05) was used as a statistical correction that controls the familywise error rate in multiple hypotheses testing for microarray studies [31]. Genes that were found to be significantly up- or down-regulated were sorted into functional groups and gene groups based on literature searches. 

### 2.6. Primer Design and qRT-PCR

Quantitative real-time polymerase chain reaction was performed on selected genes in four biological replicates using the Verso cDNA Synthesis Kit and Fast SYBR Green Master Mix following the manufacturer’s protocol and described by Musser *et al*. (2012) [29] to validate and expand the microarray data set using the same RNA sample previously isolated as described. Selected template cDNA primers were targeted at the 3' end of each gene. qRT-PCR data was analyzed by the ΔΔCt method to determine the magnitude of up- or down-regulation for each gene tested between mock and experimentally infected larvae [32]. Genes of interest were also compared against an endogenous control gene, eukaryotic initiation factor 5c (EIF5c), using the Applied Biosystems Step-One Plus qPCR system (Applied Biosystems by Life Technologies). EIF5c was selected as the endogenous control gene based on the stability of its expression from the microarray and qRT-PCR results. The primers used can be found in Table 1.

**Table 1:** Primers designed to verify gene regulation in the gut epithelium.

Gene Identifier ^1^	Gene Name	Forward Primer	Reverse Primer
AAL34109.1	Alpha-amylase	5'-accgttggcgtcaaatctac-3'	5'-ggagctgctgtagtggtcgt-3'
AAT08964.1	Cytochrome P450 (CYP)	5'-gttggatcccattcagtgct-3'	5'-ctggctcttgagatgctggt-3'
EEB16964.1	Glucose Dehydrogenase (GDH)	5'-tgactgtccaagactggtg-3'	5'-atcgctccgctctttctccta-3'
AAZ52554.1	Prophenoloxidase Subunit 2 (PROPO)	5'-gcctaccagttgttcgttat-3'	5'-agggtactttctgtccttcag-3'
AAO16241.1	Effector Caspase (ECAS)	5'-caagccagagaacctgtggt-3'	5'-gaagcagaaggtgagccatc-3'
ACL51928.1	Lysozyme (LYZ)	5'-atgagttgaggaggcaagga-3'	5'-gcccactttgtctgtcttcc-3'
ACD74811.1	Eukaryotic Initiation Factor 5c	5'-agaggttcgtcgagtggcta-3'	5'-agcctcactggctgctctta-3'

^1^ Gene identifiers correspond to the reference numbers specified by microarray analysis.

## 3. Results and Discussion

### 3.1. Ontology of Differentially-Regulated Genes in Response to Baculovirus Infection

**Figure 1:**
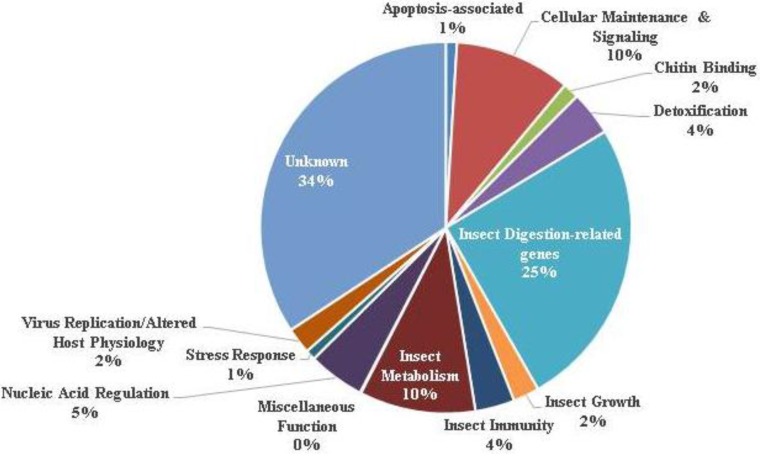
Global gene expression profile of the gut epithelium of *Helicoverpa zea* infected with HzSNPV. Percentages shown in the pie chart represent the percent of genes altered in each group out of the 1,138 genes altered in this study.

**Figure 2:**
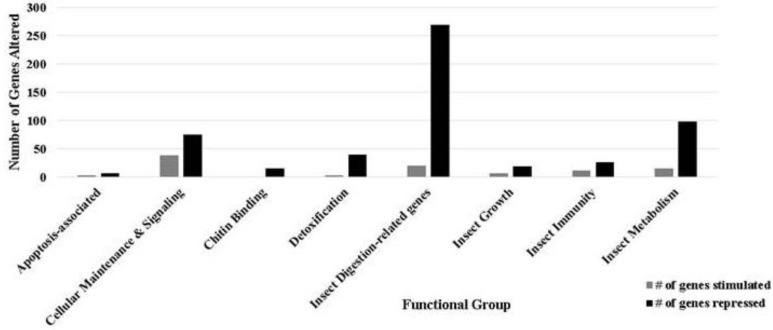
Number of genes stimulated or repressed due to viral infection associated with specific gene groups. Groups shown were most affected 24 h post infection in the gut epithelium.

The transcriptome of the gut epithelial tissue of *H. zea* infected *per os* with HzSNPV was analyzed by microarray 24 h post infection. 1,138 genes were significantly altered (*p* < 0.05) by baculovirus infection. Of the 1,138 genes, 748 genes were annotated and sorted (Figure 1 and Figure 2, Table 2 and Supplementary Table S1). Figure 1 and Figure 2 offer a survey of differentially regulated genes that were sorted based on ontology among those with defined function.

**Table 2:** Identified genes from the gut epithelium infected with HzSNPV pertaining to apoptosis, detoxification, and immunity with fold change and NCBI reference sequence indicated. The data listed represents the significant (Benjamini-Hochberg corrected *t*-test, *p <* 0.05) fold change in gene expression.

Gene classification/name	Fold change	Gene Identifier
*Apoptosis*		
Caspase short class	−1.97	XP_320581.3
CG10641-PA	2.41	XP_624640.1
Daxx-like protein	2.67	XP_973237.1
Death related ced-3/Nedd2-like protein; Initiator caspase	−1.92	NP_001108337.1
Death related ced-3/Nedd2-like protein; Initiator caspase	−1.65	NP_001108337.1
Death related ced-3/Nedd2-like protein; Initiator caspase	−1.35	NP_001108337.1
Death related ced-3/Nedd2-like protein; Initiator caspase	−1.24	NP_001108337.1
Effector caspase; SI-caspase-1	−2.57	AAO16241.1
Survivin	−1.86	XP_001662572.1
*Detoxification*		
Cytochrome P450	−8.26	XP_0010604810.1
Cytochrome P450	−6.82	XP_0010604810.1
Cytochrome P450	−3.59	XP_0010604810.1
Cytochrome P450	−16.09	NP_001104007.1
Cytochrome P450	−11.02	NP_001104007.1
Cytochrome P450 CYP4S1	−6.88	ABU88427.1
Cytochrome P450 monooxygenase	−5.41	NP_001104007.1
GA10313-PA	3.22	XP_001601512.1
Glutathione S-transferase	3.39	AAL23839.1
Glutathione S-transferase	−9.38	ABU88426.1
Glutathione S-transferase GSTX01	−11.14	ABK29516.1
Glutathione S-transferase 1	−8.69	P46430.1
*Insect Immunity*		
Adhesion-like transmembrane protein	2.61	CAB65413.1
*H. armigera* cecropin-D (HacD)	2.49	AAX51193.1
Hemolymph proteinase 18	4.41	AAZ52554.1
M-like protein	5.07	YP_313517.1
Prophenoloxidase subunit 2	5.46	ABF18489.1
Toll-interacting protein	1.66	XP_975168.1

### 3.2. Apoptosis

Apoptosis-related transcripts in the gut epithelium represented <1% (11 genes) (Table 2 and Table S1). HzSNPV infection altered these groups by down-regulating more than half (7) of those identified as differentially regulated.

### 3.3. Cellular Maintenance and Signaling

10% (115) of the genes shown to be differentially regulated in response to baculovirus infection related to cellular function, and of these, approximately 2/3 were down-regulated (Figure 2, Table 2 and Table S1). These included genes such as cyclin and cyclin-activating kinases, transporters and cell membrane constituents, as well as those with defined roles in signal transduction such as chemosensory proteins. Genes responsible for cell maintenance and signaling constituted 69% (67) of the genes. The remaining genes related to energy metabolism were almost universally down-regulated (25 genes or 96%).

### 3.4. Chitin Binding

Following infection, 16 genes (100%) associated with chitin binding were found to be substantially repressed following baculovirus infection (Table S1). Chitin deacetylase, chitin binding domains, cuticle proteins, and peritrophic membrane genes were repressed due to viral infection. The peritrophic matrix is present, particularly in Lepidopterans, to serve as a barrier to crystalline toxins, various anti-nutritive enzymes, and pathogenic infections. However, through a long evolutionary history with their hosts, baculoviruses including, but not limited to HzSNPV, have successfully acquired genes through horizontal gene transfer [33]. Baculoviruses were able to obtain genes like chitinase, which is an enzyme that breaks down chitin and is a main constituent of the peritrophic matrix [33]. Two genes (Table S1) coding for insect intestinal mucin 3, responsible for gut chitin binding and the regeneration of damaged areas of the peritrophic compartment, were found to be down-regulated approximately 4 and 6 fold, respectively, during infection. Poor membrane integrity may allow the virus to translocate more efficiently from the gut within the host.

### 3.5. Detoxification

Two major classes of detoxification enzymes (Table 2 and Table S1) were altered following infection, specifically cytochrome p450’s (CYP) and glutathione S-transferases (GST). 90.9% (40 genes) in the gut epithelium were down-regulated. 28 CYP genes ranged from a 3.9 fold increase to a down-regulation of 23.2. Infection also led to the suppression of 3 GST-related genes between 8 and 11 fold, and the induction of one by approximately 3-fold. Other genes broadly relating to detoxification were down-regulated including alkaline phosphatase 1 and 2, and one gene for hemolysin-type calcium-binding protein (Table S1).

Our array results further argue for the production or presence of cytotoxic compounds by 24 h post infection. Some baculoviruses produce conotoxin-like compounds, which are disulfide-rich ion channel antagonists [34,35,36]. These are toxin homologs that act on ion channels and inhibit proper function of the various sodium, potassium, calcium, and/or voltage-dependent ion channels, which are supported by the noted up-regulation of various ion transporters described in the cellular function section. Another possible explanation for the presence of detoxification-related compounds such as cytochrome p450 and GST is viral-mediated breakdown of cellular components, such as the endocytic vesicles, or other cytolytic activities prompting such a response from either affected or neighboring cells.

It has been suggested that cytochrome P450’s are responsible for toxin degradation and general detoxification, whereas glutathione S-transferase genes are responsible for intracellular detoxification and aid in the resistance of insecticides [37,38]. Cytochrome P450’s, in generalist polyphagous insects like *H. zea*, are speculated to have very broad ranges of specificity for environmental and microbial resistance. Not only can these insects feed on many plants, but the series of cytochrome P450’s allows them to resist microbial toxins, which include conotoxins and conotoxin-like homologs. The class of glutathione S-transferase genes is associated with the degradation of insecticides and is efficient at detoxifying pyrethroids [38]. The gut epithelium has several genes that function in intracellular detoxification such as glutathione S-transferase; broad down-regulation among these could potentially be attributed to the virus antagonizing various cell transporters during infection.

### 3.6. Digestive Function

A large number of genes relating to insect metabolism and digestion were significantly altered in response to viral infection. Of the 289 differentially regulated genes in the digestive category (Table S1), the vast majority (93%) were down-regulated. 128 genes that code for proteases and protein-turnover-related proteins were among those significantly different, whereas 52 play known roles in carbohydrate utilization. Ten percent of those genes identified as differentially regulated were related to insect metabolism; these exhibited a similar trend of down-regulation in response to baculovirus infection. Among these metabolism related genes were those including various dehydrogenases, lyases, and lipases associated with sugar, amino acid, and fatty acid synthesis. Overall digestive and metabolic function of the gut epithelium was considerably affected by viral infection (Figure 1), which showed a combined 90% down-regulation among those genes that were differentially regulated in response to viral infection. The large number of genes altered in the gut is likely indicative of this tissue as a site of primary infection. These results, demonstrating a breadth of the digestion-related genes, differ from those of Breitenbach *et al.*, which may reflect the different tissue (hemocytes *versus* gut epithelium) used in that study, differences in timing (12 *versus* 24 h), or a combination thereof [20]. 

### 3.7. Immunity

Of the identified differentially-regulated genes, 4% (39 genes) of these have been implicated in insect immunity (Table 2 and Table S1). The majority (69%) of these immunity-related transcripts were shown to be repressed in response to baculovirus infection. Important genes for humoral immunity were both up- and down-regulated. Innate and humoral immunity, specifically antibacterial protein and antimicrobial peptide binding genes, were repressed while antiviral genes prophenoloxidase and *Helicoverpa armigera* cecropin-D (HacD) were up-regulated. Toll pathway genes, surface antigen ariel1 genes, and hemolymph protease genes, all of which are immune reactive genes, were stimulated over 2.2 fold in the gut. Genes for encapsulation were found to be alternatively up- or down-regulated, depending on the specified transcript.

Insects mount an immune response to pathogens that shares limited similarities with that of mammals. We observed a pronounced down-regulation of genes in the gut associated with immunity and the host response to pathogens. Again, this is due to the site of infection in synergy with the time point. In insects, the largest portion of the immunity-related genes that target viruses is located in the gut epithelium, where an insect can rid itself of a virus by activating pro-apoptotic signaling, thus passing apoptotic cells and vesicles through the digestive tract [39]. The gut acts as the center for signaling cascades that can induce further immune response throughout the organism in order to ensure stability of the gut for digestion, as well as ensure survival of the organism [39]. These findings broadly agree with those of Nguyen, *et al.* who documented alterations of those genes involved in the TOLL signaling pathway, caspases, and adhesion-related genes [27]. Additionally, studies by Shelby and Popham in 2008 showed the alteration of gram negative bacteria recognition proteins, as well as, the alteration in macrophage migration inhibitory factors, both of which are seen here [40]. 

### 3.8. Virus Related Replication/Physiology Alteration/Endogenous Retroviral Intrusions

Differentially expressed transcripts associated with endogenous retroviral intrusions (errantiviruses) or retro-transposon-like elements were detected in the microarray analysis, as was a selection of host genes that are thought to be exploited by the virus to facilitate its replication cycle (Table S1). A number of genes involved in insect development and life cycle changes were differentially transcribed, as has been well documented in prior reports [20,27]. Half of these genes (54%, or 14 genes) were up-regulated following infection. Among the host genes associated with viral replication that were differentially regulated were DEAD H- and X-Box helicases, ribonucleoside-diphosphate reductase large chain, and retroelement polyproteins. Several genes responsible for baculovirus-mediated suppression of ecdysteroids and encoding baculovirus-specific chitin-binding proteins represented 10 of these genes, or 1.3%.

### 3.9. Quantitative Real Time-Polymerase Chain Reaction

**Figure 3:**
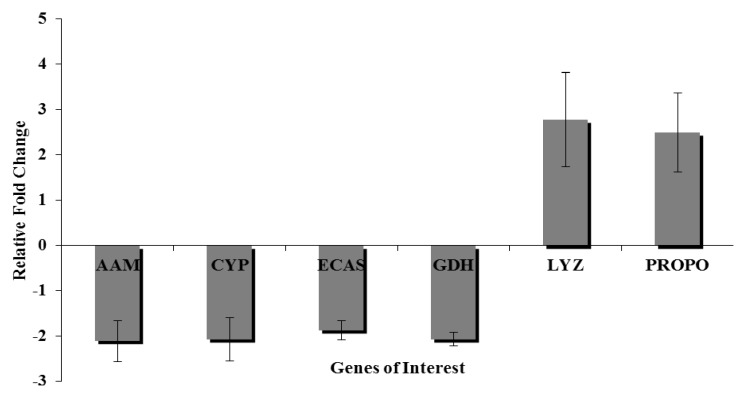
Relative fold changes (y-axis) in expression of alpha-amylase (AAM), cytochrome P450 (CYP), effector caspase (ECAS), glucose dehydrogenase (GDH), lysozyme (LYZ), and prophenoloxidase (PROPO). These data indicate the relationship between these genes and the fold change expressed between mock and virus infected treatments.

Several genes of interest were analyzed by qRT-PCR in order to validate and extend response in gut epithelium to viral infection (Figure 3). The data confirm transcriptional repression of alpha amylase, cytochrome P450, effector caspase, and glucose dehydrogenase, while prophenoloxidase was stimulated, also in agreement with the DNA microarray data (*p* < 0.05, Benjamini-Hochberg).

## 4. Conclusions

The transcriptional profile of *H. zea* gut epithelial tissue was analyzed by DNA microarray 24 h following viral infection and compared to that of uninfected tissues. The gut epithelium is a tissue that, by virtue of its function, undergoes dynamic changes in transcription during different life stages, diet and feeding conditions, and exposure to environmental hazards such as pathogens or toxins [41,42,43]. It is generally accepted that the gut is the site of primary baculovirus infection, and our analyses reveal a rapidly evolving reaction by the host to respond offensively to the virus, as well as virus manipulation of the host defense and cellular machinery to propagate itself at the expense of the insect [44,45,46]. These findings are supported by those seen by Breitenbach *et al.* [20] and Nguyen *et al.* [27], where both analyzed *Heliothis virescens* or *H. zea* transcriptional responses to similar baculoviruses, HzSNPV or *Helicoverpa armigera* nucleopolyhedrovirus (HearNPV), respectively. Importantly, as well as being the first report on *H. zea* gut global transcription in response to baculovirus infection, these data complement those of the other groups, who focused on hemocytes in *H. virescens* or cell line cultures of *H. zea*, and careful comparison may provide insights into how these vastly different tissues both respond to, and are exploited by, the replicating virus.

In agreement with extensive research in baculovirus-mediated suppression of the insect’s apoptotic pathway(s), our results likewise demonstrate the extent and magnitude of the effect of baculovirus inhibitors of apoptosis [19,47,48,49,50]. Over half of the apoptotic genes analyzed were suppressed; some genes related to apoptosis, however, were up-regulated. Most notably, in the gut epithelium (Table 2) short class caspase, initiator caspase (DREDD), and effector caspase were all repressed in response to viral infection. Baculoviruses, including HzSNPV, inhibit the efficacy of the caspase, which are enzymes that promote the apoptotic program of planned cell death. Survivin (Table 2) works in tandem with viral-encoded apoptotic inhibitors as it acts as an inhibitor of apoptosis proteins (iaps) [51,52]. While the vast majority of the genes in this group for the gut epithelium were repressed, we detected a number that were up-regulated, such as DAXX-like protein. While it remains incompletely characterized, this protein is thought to play a crucial role in cell survival signaling [53,54,55]. Therefore, the higher expression levels of DAXX-like protein observed in this study suggests the stimulation of the anti-apoptosis regulation, which in turn, prolongs the infectivity of HzSNPV throughout its infection cycles. The observation of a number of up-regulated genes related to apoptosis likely points to the relatively early 24 h time point at which these tissues were harvested; the general outcome and hallmark of lethal infection is a global suppression of the apoptotic response, allowing the virus to disseminate within and without the hemoceol by later times post infection. Our work also reinforces the extent to which the virus modulates the life cycle and development of the insect during its natural history of infection, demonstrating hormone suppression, specifically found in phenol UDP-glucosyltransferase (Table 2 and Table S1), that functions to suppress ecdysteroids [56,57]. This functionally slows, or stops the progression of molting/pupating, thus allowing the virus to prolong infection and produce more environmentally stable polyhedra. Other notable genes involved in viral exploitation of host function include DEAD Box RNA helicase genes, which aid in viral replication [58,59]. HzSNPV, as well as other baculoviruses, alter eukaryotic initiation factors in the cells they manipulate [60,61]. Also of significance was the observation of a generalized inhibition of several key metabolic pathways that function in energy storage and utilization. Whereas healthy larvae move metabolites and digestive products efficiently from the midgut into the hemocel for storage in the fat body, the nearly global inhibition of these processes, including glucose and other sugar utilization, fatty acid metabolism, and amino acid trafficking offer additional insights as to the degree to which baculoviruses alter host behavior at the molecular level to promote their replication and spread at the expense of the host.

Endogenous retrotransposon and retrovirus-like genes were detected in this microarray study. These genes are related to the evolutionary histories of insects and retroviral elements that have been assimilated into the host genome [62,63]. These genes generally code for gag-pol repeat proteins, as well those identified as retroelement polypeptides and reverse transcriptase, findings that agree with previous analysis of other tissues in infected HzSNPV [20]. It remains poorly understood how baculovirus infection stimulates retroviral reactivation and/or retrotransposon activity; given the large number of transposable elements found in these and other insects, it is quite likely that a combination of direct stimulation by baculovirus transcriptional transactivators and cellular transactivators in the infected cells both play a role in mobilizing these otherwise quiescent elements [64].

Taken together, these results demonstrate the degree to which lethal HzSNPV infection modulates the gut epithelium of its natural host, and adds to an evolving picture of virus-mediated transcriptional regulation during the natural history of infection by baculoviruses. Our real-time qRT-PCR results confirm the validity of a sampling of those genes that were identified in our microarray, and further analysis of baculovirus transcriptional targets may provide new insights as to how this and closely related baculoviruses have evolved to exploit host machinery, as well as identify cellular pathways and effector molecules that may suppress replication and allow for host escape from mortal infection.

## Supplementary Files

Supplementary File 1
